# Case Report: Severe Acute Pulmonary COVID-19 in a Teenager Post Autologous Hematopoietic Stem Cell Transplant

**DOI:** 10.3389/fped.2022.809061

**Published:** 2022-03-03

**Authors:** Fabian J. S. van der Velden, Frederik van Delft, Stephen Owens, Judit Llevadias, Michael McKean, Lindsey Pulford, Yusri Taha, Grace Williamson, Quentin Campbell-Hewson, Sophie Hambleton, Rebecca Payne, Christopher Duncan, Catriona Johnston, Jarmila Spegarova, Marieke Emonts

**Affiliations:** ^1^Paediatric Immunology, Infectious Diseases and Allergy, Great North Children's Hospital, Newcastle upon Tyne Hospitals NHS Foundation Trust, Newcastle upon Tyne, United Kingdom; ^2^Faculty of Medical Sciences, Translational and Clinical Research Institute, Newcastle University, Newcastle upon Tyne, United Kingdom; ^3^Paediatric Oncology, Great North Children's Hospital, Newcastle upon Tyne Hospitals NHS Foundation Trust, Newcastle upon Tyne, United Kingdom; ^4^Paediatric Intensive Care, Freeman Hospital, Newcastle upon Tyne Hospitals NHS Foundation Trust, Newcastle upon Tyne, United Kingdom; ^5^Paediatric Respiratory Department, Great North Children's Hospital, Newcastle upon Tyne Hospitals NHS Foundation Trust, Newcastle upon Tyne, United Kingdom; ^6^Virology, Newcastle upon Tyne Hospitals NHS Foundation Trust, Newcastle upon Tyne, United Kingdom; ^7^Paediatric Intensive Care, Great North Children's Hospital, Newcastle upon Tyne Hospitals NHS Foundation Trust, Newcastle upon Tyne, United Kingdom; ^8^Infectious Disease and Tropical Medicine, Newcastle upon Tyne Hospitals NHS Foundation Trust, Newcastle upon Tyne, United Kingdom; ^9^Pharmacy, Newcastle upon Tyne Hospitals NHS Foundation Trust, Newcastle upon Tyne, United Kingdom

**Keywords:** pediatric, COVID-19, stem cell transplant, immunomodulation, immunodeficient

## Abstract

Pulmonary severe acute respiratory syndrome coronavirus 2 (SARS-CoV-2) infection in children is generally described as mild, and SARS-CoV-2 infection in immunocompromised children are observed as generally mild as well. A small proportion of pediatric patients will become critically ill due to (cardio)respiratory failure and require intensive care treatment. We report the case of a teenager with Hodgkin's lymphoma who acquired SARS-CoV-2 (detected by PCR) on the day of her autologous stem cell transplant and developed acute respiratory distress syndrome, successfully treated with a combination of antivirals, immunomodulation with steroids and biologicals, and ECMO.

## Introduction

Primary pulmonary coronavirus disease 2019 (COVID-19) in children is generally regarded as a mild infection (1). Severe acute respiratory syndrome coronavirus 2 (SARS-CoV-2) in respiratory droplets directly infects susceptible cells via angiotensin-converting enzyme 2 and transmembrane protease, serine 2 receptors expressed in the respiratory tract and other organs, subsequently causing illness by eliciting an aberrant host response (2). In a minority, a post-COVID-19 inflammatory syndrome is observed (3, 4). Treatment consists of supportive management, and if the patient requires supplemental oxygen, dexamethasone, remdesivir, and SARS-CoV-2 monoclonal antibodies in eligible patients are considered (5).

COVID-19 has rarely been reported in immunocompromised children, including hematopoietic stem cell transplant (HSCT) recipients, and is generally mild (6–8). These children were more likely to develop a severe inflammatory syndrome with SARS-CoV-2 months after HSCT than in early post-transplant. This prompts host response after immune reconstitution as an important determinant for complications and suggests that those who are immunocompromised might be relatively protected, yet numbers are low (9).

COVID-19 can cause severe respiratory failure due to alveolar damage (10). Extracorporeal membrane oxygenation (ECMO) has been successfully used in patients with severe cardiopulmonary failure secondary to COVID-19, refractory to conventional therapies (11, 12).

Guidance for management of suspected COVID-19 recommends venovenous (VV)-ECMO for eligible patients with COVID-19 acute respiratory distress syndrome (ARDS) (13). In immunocompromised patients with viral pneumonia, ECMO is controversial, due to increased mortality (14). Eligibility depends on institutional protocols (14).

We describe the case of a teenager with Hodgkin's lymphoma who received curative high-dose chemotherapy with autologous HSCT and tested positive for SARS-CoV-2 by PCR on the day of transplant. She developed ARDS, successfully treated with a combination of antivirals, immunomodulation with steroids and biologicals, and ECMO.

## Case Description

**Figure 1:**
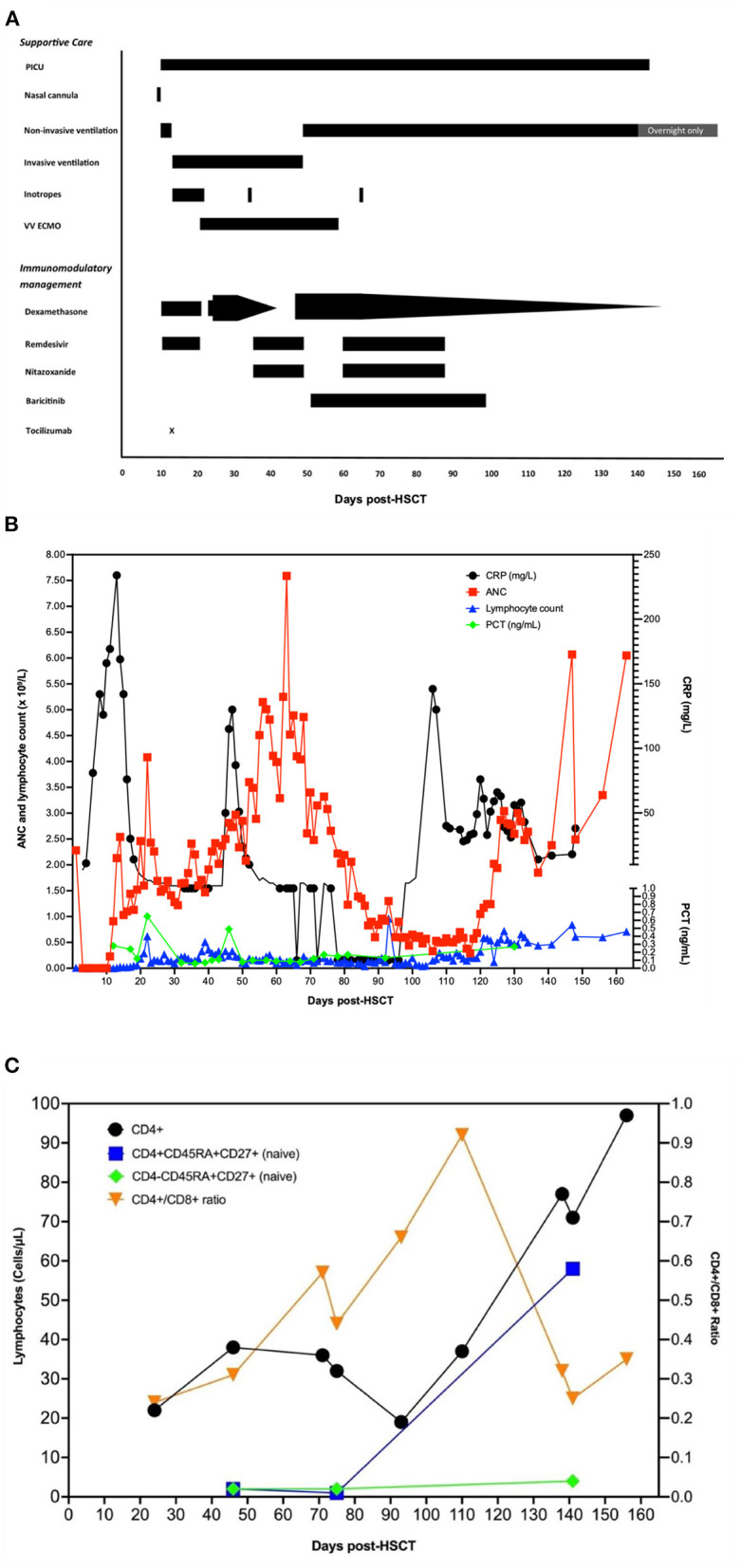
A schematic overview of respiratory and immunomodulatory management over time. **(A)** Overview of respiratory support and medical interventions over time. Day 0 is the day of autologous stem cell infusion and the day the patient first tested positive for SARS-CoV-2. **(B)** Overview of inflammatory markers and neutrophil and lymphocyte counts over time. Day 0 is the day of autologous stem cell infusion. ETT/BAL, endotracheal/bronchoalveolar lavage secretions; NTS, nose–throat swab; ANC, absolute neutrophil count; CRP, C-reactive protein; PCT, procalcitonin. **(C)** CD4 lymphocytes and naïve T-cell counts over time. Day 0 is the day of autologous stem cell infusion.

A 14-year-old patient with relapsed Hodgkin's lymphoma received curative high-dose carmustine/etoposide/cytarabine/melphalan chemotherapy and autologous HSCT. On the day of the stem cell infusion (day 0), she asymptomatically tested as polymerase chain reaction (PCR) positive for SARS-CoV-2 on contact screening. A schematic overview of respiratory and immunomodulatory management over time is given in Figure 1.

**Figure 2:**
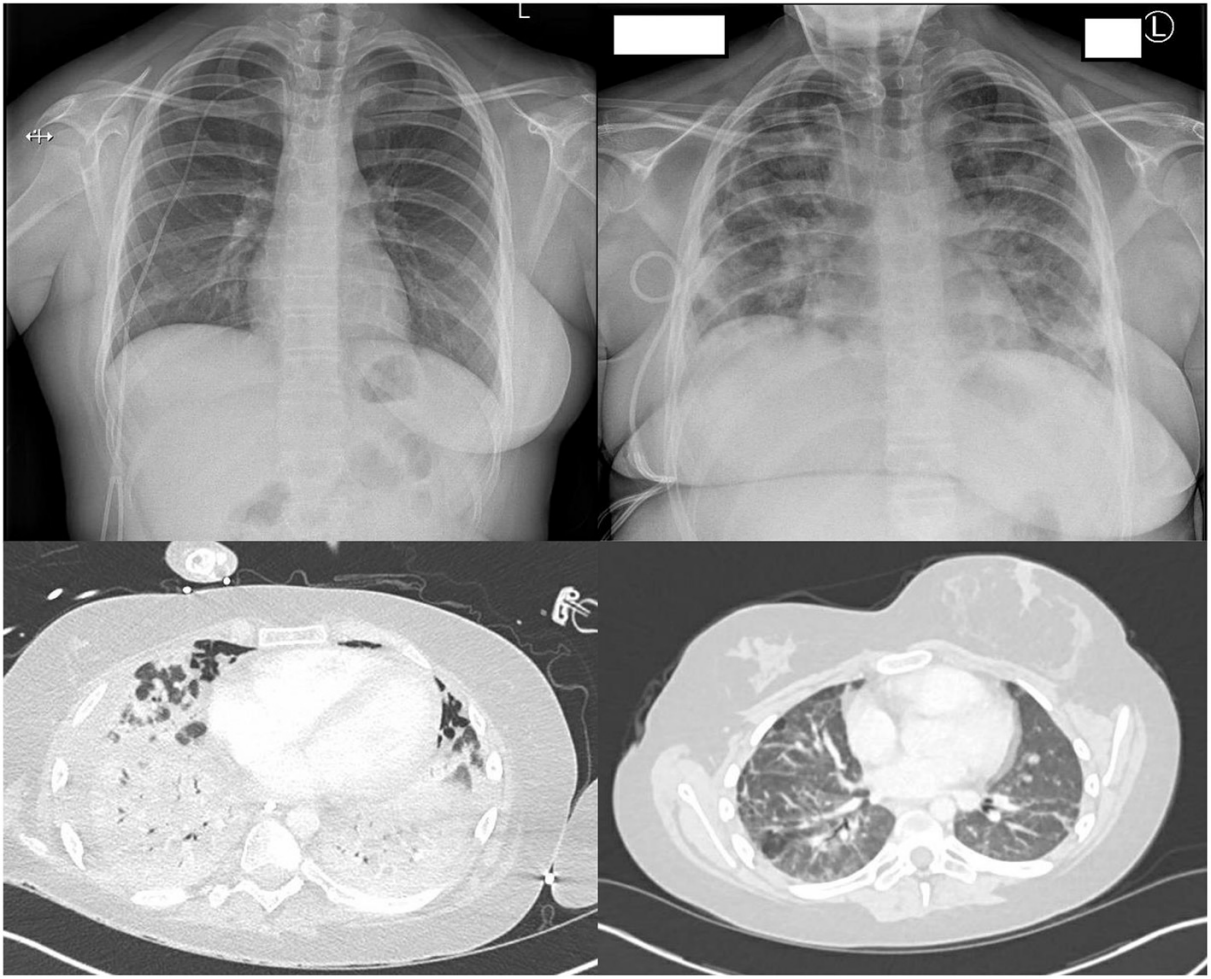
Chest radiology during admission. Top left: Chest X-ray at baseline (Day −1). Top right: Chest X-ray at ARDS development (Day +10). Bottom left: CT thorax post-tracheostomy (Day +47) showing extensive inflammation fitting with COVID-19. Bottom right: CT thorax 3 months post discharge showing residual lung changes.

Ten days later, around neutrophil engraftment, she became unwell with fever, respiratory distress, and elevated inflammatory markers (C-reactive protein 142 mg/L). Upon cardiorespiratory deterioration, she was transferred to the pediatric intensive care unit (PICU) for mechanical ventilation and inotropic support. Echocardiography was normal. She was given a course of dexamethasone (6 mg/day intravenously (IV) once daily for 10 days) and remdesivir (200 mg loading dose, followed by 100 mg IV once daily, total 10 days) for COVID-19 ARDS and IV antibiotics for potential concurrent serious bacterial infection (SBI) and piperacillin–tazobactam 4.5 g 6 hourly for 5 days and then switched to meropenem 2 g 8 hourly for 14 days, concurrent with teicoplanin 12 mg/kg/day. Tocilizumab was initially withheld until SBI control on day +14. Other non-infectious etiologies were considered. Peri-engraftment respiratory distress syndrome (PERDS) was considered to play a role, as she was engrafting at time of deterioration and expected to respond to steroid therapy to reduce inflammation. There was no evidence of active hemorrhaging, no blood in sputum, nor abnormal coagulation, making diffuse alveolar hemorrhage unlikely. Imaging was consistent with COVID-19 pneumonia (Figure 2).

Despite treatment, she remained SARS-CoV-2 positive on biweekly PCRs and had further cardiorespiratory deterioration. She remained lymphopenic, and inflammatory markers were low (Figure 1C).

After discussion between infectious diseases, PICU, and oncology, she was deemed eligible for VV-ECMO, given progressive respiratory failure with maximized adjuvant therapies. Higher mortality risk associated with profound immunosuppression was weighted against favorable COVID-19 disease outcome in younger patients, single-organ failure, expected immune reconstitution post-HSCT, and curative lymphoma treatment. She was cannulated for ECMO on day +20.

Having persistent low SARS-CoV-2 Ct values, a surrogate marker for high viral load caused by unrestrained viral replication (Supplementary Data 2), a second course of IV remdesivir (200 mg loading dose followed by 100 mg once daily for 14 days) was given and IV dexamethasone recommenced (13.2 mg/day in two doses for 10 days and subsequently weaned off at 1 mg/day) (Figure 1A). Nitazoxanide (500 mg IV 12 hourly for 14 days) was experimentally added, due to its potential to inhibit viral replication and promote balance between pro- and anti-inflammatory responses (15). The local virology laboratory employs a myriad of SARS-CoV-2 assays, nucleic acid extraction systems, and cyclers (Supplementary Data 2). Regardless of the PCR methodology used, no significant discrepancy was observed between results of different respiratory samples with consistent trends seen throughout the course of the illness.

On day +46, tracheostomy was performed for an expected prolonged course on ECMO and ventilation, after which she unexpectedly had pulmonary deterioration. A CT scan (Figure 2) showed extensive parenchymal deterioration with diffuse dense consolidation, suggesting COVID-19 progression, despite two negative SARS-CoV-2 PCRs. She was treated for suspected SBI with meropenem, teicoplanin, and linezolid for 14 days, as her procalcitonin was slightly elevated (0.49 ng/ml). Bronchoalveolar lavage showed extensive inflammation and no hemorrhage, and viral, fungal, and bacterial investigations were negative. Echocardiography was not suggestive of cardiac pathology. Dexamethasone was restarted (13.2 mg IV once daily for 21 days, followed by 0.5 mg/48 h weaning), and other options for immunomodulation were explored, ideally steroid sparing to aid immune reconstitution, yet keeping lung inflammation under control. Baricitinib, which has a shorter half-life than tocilizumab, was started (4 mg/day, enteral for 48 days).

One week after completing her second course of remdesivir and nitazoxanide, she became SARS-CoV-2 PCR positive again; both were restarted using the same dosage as previous courses.

Clinically she improved, and ECMO was stopped after 39 days (day +57), after a stable ECMO run without major complications. The following weeks she continued to improve; remdesivir and nitazoxanide were stopped after 2 weeks of being SARS-CoV-2 PCR negative (a course of 29 days for both drugs), baricitinib was stopped a few weeks later (day +98, total duration 48 days), and she continued to be weaned off dexamethasone. Discharge to the long-term ventilation ward for rehabilitation occurred after 132 days on PICU (day +143). She made great progress with rehabilitation in the subsequent months. Ventilation support ceased on day +195. She still has a reduced lung function but was fit for discharge to her local hospital for further rehabilitation on day +218.

## Methods

Informed consent and assent for publication were obtained from the legal guardian and patient. Data were collected from the hospital clinical records.

Immunological fluorophenotyping analyses of T-lymphocyte response to specific SARS-CoV-2 spike peptides were performed by the comparison of peripheral blood mononuclear cells (PBMCs) from our patient and six PBMC control from seropositive SARS-CoV-2 IgG healthcare workers >18 years of age and participating in the PITCH study. The detailed functional immunology analysis methodology can be found in Supplementary Data 1.

## Results

### Virology

SARS-CoV-2 RNA shedding remained high for 43 days, as indicated by low/early PCR Ct values in bronchoalveolar lavage, tracheal aspirate, and nose–throat swabs. Tracheal aspirate results obtained by an Altona RealStar® SARS-CoV-2 RT-PCR Kit 1.0 were used as the primary guide for clinical decision, while virus clearance was ascertained in all upper- and lower-respiratory-tract samples. A temporary decline in virus shedding with negative PCR, noted during re-treatment with remdesivir, slowly reversed with worsening Ct values after stopping the antiviral agent. Following restart of remdesivir, Ct values gradually improved with PCR tests turning negative in ET secretions by day +61 and nose–throat swabs by day +72. SARS-CoV-2 PCRs remained negative for the following 120 days.

### Immune Reconstitution

Neutrophil reconstitution occurred on day +12 post-HSCT. Lymphocyte subsets were determined monthly from day +45, and lymphocyte reconstitution was observed on day +120. Naïve T lymphocytes started to occur from day +140 (Figure 1C).

#### Patient T Lymphocytes Showed Antigen-Specific *ex vivo* Responses to SARS-CoV-2 Peptides

**Figure 3:**
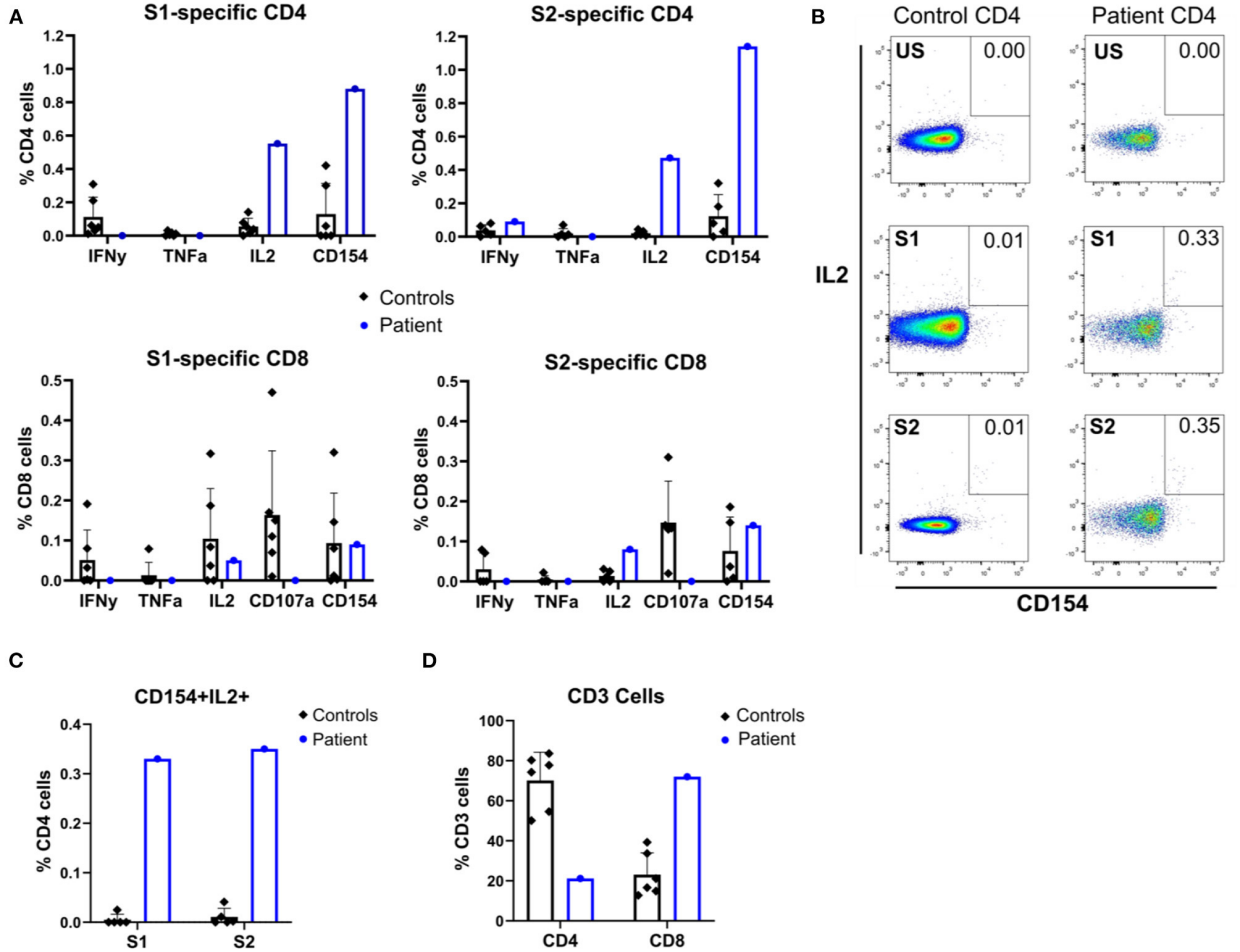
Flow cytometric immunophenotyping reveals SARS-CoV-2 peptide-specific responses of patient and seropositive control T cells. **(A)** Patient and control seropositive PBMCs were stimulated with SARS-CoV-2 S1 or S2 peptide pools before flow cytometric assessment for induction of cytokines (IFNγ, TNFα, and IL-2) and surface markers of activation (CD154 and CD107a). **(B)** Representative flow cytometry dot plots showing expression of CD154 and IL-2 by stimulated patient or control PBMC, gated on CD4+ T cells. US, unstimulated; S1, spike protein 1; S2, spike protein 2. **(C)** Quantification of double-positive CD154+IL2+ CD4 cells after stimulation with spike peptide pools S1 and S2. **(D)** CD4 and CD8 cell counts.

Despite low CD4-lymphocyte counts in the patient (CD4/CD8 ratio 0.29, Figure 1C) and *in vivo* exposure to corticosteroids and JAK inhibitors, antigen-specific responses were clearly present. Proportionally, the patient's CD4-T-lymphocyte responses were larger than CD8-T-lymphocyte responses, and no upregulation of CD107a (LAMP-1) to indicate antigen-specific degranulation of the latter was seen (Figure 3A). Compared with that in healthy seropositive controls, a higher proportion of patient CD4-lymphocytes produced IL-2 and/or underwent activation (judged by CD154 (CD40L) upregulation) in response to S1 and S2 peptide pools (Figures 3B–D). Nonetheless, patient T lymphocytes showed poor production of IFNγ and TNFα in response to S1 or S2 spike peptides (Figure 3A).

SARS-CoV-2-specific antibodies, negative on day +11, were positive on day +190. The patient did not receive any immunoglobulins during her admission. Samples were analyzed using the Roche Elecsys Anti-SARS-CoV-2 assay, according to the manufacturer's instructions.

## Discussion

We describe the first pediatric patient with COVID-19 ARDS acquired immediately at the time of HSCT, successfully managed with ECMO, antivirals, and immunomodulation. We show SARS-CoV-2-specific cellular and humoral immune response development, despite ongoing immunosuppression.

Lymphopenia and specific CD4-lymphocyte response are observed in SARS-CoV-2 infection and appear to correlate with severity (16). Our patient tested positive on day 0 of HSCT, and T-lymphocyte reconstitution is not expected until 3 months post-transplant. Viral clearance is associated with T-lymphocyte reconstitution and naïve T-lymphocyte development (17). Pneumonitis in HSCT patients has an increased mortality risk (18).

We observed minor discrepancies between SARS-CoV-2 Ct values in nose–throat swab samples and bronchoalveolar lavage samples. Different sample sites and volumes can explain this, and it has been observed in immunocompetent patients that viral loads peak on average 2 weeks after symptom onset in lower-respiratory-tract samples, whereas viral loads decrease on average 1 week after symptom onset in the upper respiratory tract (19, 20). When available, clinical decisions were based on sample positivity, using Ct values as a guide of viral activity, as we suspected clearing SARS-CoV-2 from the lungs would take longer due to prolonged immunosuppression and clinical condition.

Although COVID-19 ARDS was most likely, we considered other non-infectious etiologies during admission. PERDS occurs in 2.5% of autologous HSCT (21), and as the patient's deterioration happened during engraftment, it is reasonable to assume that it played a role in her clinical condition and inflammation during her stay in the PICU. PERDS is also treated with systemic steroids, which makes it difficult to estimate how large the role of engraftment was. Post neutrophil engraftment, it would not explain her subsequent episodes of deterioration well enough in the light of continuously low SARS-CoV-2 Ct values, which made COVID-19 ARDS our overarching diagnosis. There were no issues with coagulation, nor evidence of diffuse alveolar hemorrhage or pulmonary embolisms on imaging. Another known pulmonary complication of HSCT is cryptogenic organizing pneumonia; however, this occurs at a later stage post-transplant, median 108 days after HSCT (22). Additionally, imaging did not show characteristic changes associated with cryptogenic organizing pneumonia (23).

Dexamethasone reduced inflammation well but delayed immune reconstitution required for virus clearance. A careful balance between immunosuppression for controlled immune reconstitution and limitation of inflammation potentially causing pulmonary damage was essential (24). To aid steroid weaning, baricitinib was chosen as an immunosuppressant. It inhibits the cellular signaling pathway of cytokines elevated in severe COVID-19 and acts against SARS-CoV-2 cellular entry (25, 26). It was chosen over tocilizumab in this ongoing immunodeficient patient because (1) tocilizumab significantly increases the risk of fungal infection and (2) the shorter half-life of baricitinib allowed prompt cessation in case of suspected superinfection. A double-blind, placebo-controlled randomized trial in adults showed that 14 days of baricitinib plus remdesivir accelerated clinical improvement compared to remdesivir alone in patients on high-flow/non-invasive ventilation, although this was not proven in their group of 111 patients on mechanical ventilation or ECMO (27). Beigel et al. showed similar results for remdesivir vs. placebo, with remdesivir reducing time to recovery in hospitalized adults, but could not prove this in the subgroup on mechanical ventilation or ECMO (28). After careful consideration, the potential benefits of baricitinib and remdesivir outweighed the risks, despite its efficacy not being proven in adult patients on mechanical ventilation or ECMO.

Prolonged immunosuppression combined with remdesivir and nitazoxanide for SARS-CoV-2 in our patient was chosen because (1) respiratory deterioration after standard course cessation and (2) immune reconstitution post-HSCT in patients with infection are known to be associated with significant inflammation (29).

In addition to pharmaceutical interventions, we acknowledge that other factors contributed to her recovery and SARS-CoV-2 clearance. Our elected management provided us with enough time to slowly reduce steroids and allow for T-lymphocyte engraftment, while keeping associated inflammation under control. Subsequently, this enabled her immune system to clear the virus aided by pharmaceuticals.

In pediatric COVID-19, ECMO use is seldom described (11, 30, 31). In adult COVID-19, a 44–53% survival rate on ECMO was described (32, 33). It was believed that respiratory support on ECMO while her immune system recovered was our patient's only chance of survival.

In an HSCT recipient, a combination of antiviral and immunosuppressive therapies can be required to allow viral and inflammation control in pulmonary COVID-19. VV-ECMO is a potential option. Management should happen in a multidisciplinary approach, allowing optimal management in a rapidly changing field.

## Data Availability Statement

The original contributions presented in the study are included in the article/Supplementary Material, further inquiries can be directed to the corresponding author.

## Ethics Statement

Ethical review and approval was not required for the study on human participants in accordance with the local legislation and institutional requirements. Written informed consent to participate in this study was provided by the participants' legal guardian/next of kin. Written informed consent was obtained from the individual(s) and minor(s)' legal guardian/next of kin, for the publication of any potentially identifiable images or data included in this article.

## Author Contributions

FV, ME, FD, SO, CD, MM, and JL wrote the manuscript. YT conducted and interpreted the viral loads from different samples. QC-H and FD were responsible for the HSCT. SH, CD, RP, and JS were responsible for the immunological assays and interpretation. JL, LP, and GW were responsible for the PICU admission. CJ was co-responsible for medication regulatory administration. All co-authors revised the manuscript and agreed with submission.

## Funding

ME and FV are funded by European Union's Horizon 2020 research and innovation program under Grant Agreement No. 848196. The PITCH (Protective Immunity from T cells to COVID-19 in Health workers) Consortium was funded by the UK Department of Health and Social Care, with a contribution from the UK Coronavirus Immunology Consortium (UK-CIC).

## Conflict of Interest

The authors declare that the research was conducted in the absence of any commercial or financial relationships that could be construed as a potential conflict of interest.

## Publisher's Note

All claims expressed in this article are solely those of the authors and do not necessarily represent those of their affiliated organizations, or those of the publisher, the editors and the reviewers. Any product that may be evaluated in this article, or claim that may be made by its manufacturer, is not guaranteed or endorsed by the publisher.
