# Supplementary material for: Case Report: Severe Acute Pulmonary COVID-19 in a Teenager Post Autologous Hematopoietic Stem Cell Transplant
Source: Front Pediatr. 2022 Mar 3;10:809061. doi: 10.3389/fped.2022.809061 (PMC8927762; doi:10.3389/fped.2022.809061)
Supplement: Supplementary file 1 [file Data_Sheet_1.DOCX]

**Supplementary Data**

Supplementary Data 1

***Patient consent and sample collection***

Control PBMC samples were collected from healthcare workers aged 18 or over and seropositive for SARS-CoV-2 IgG, enrolled in the PITCH substudy of the Public Health England SIREN study at the Newcastle upon Tyne Hospitals NHS Foundation Trust. The SIREN study was approved by the Berkshire Research Ethics Committee, Health Research 250 Authority (IRAS ID 284460, REC reference 20/SC/0230), with PITCH recognised as a substudy on 2 December 2020. SIREN is registered with ISRCTN (Trial ID:252 ISRCTN11041050).

***Re-stimulation of PBMC with SARS-CoV-2 peptide pools***

PBMCs were isolated from blood samples using Lymphoprep (StemCell Technologies, 07851) density gradient centrifugation as per manufacturer’s instructions. Purified PMBC were plated at 1-1.5 x 10^6^ cells/well in pre-warmed RPMI-1640 medium (Sigma Aldrich, R0883) supplemented with 10% (v/v) FCS (Gibco, 10270-106), 1% (v/v) Penicillin/Streptomycin (100 U/mL and 100 μg/mL respectively; Sigma Aldrich, P0781), 1% (v/v) L-Glutamine (2 mM; Sigma Aldrich, G7513), DNAse (1ug/ml, Merck, 10104159001), and co-stimulatory antibodies CD49d (1ug/ml, Biolegend, 304339) and CD28 (1ug/ml, Biolegend, 302914) in U-bottomed 96-well plate, to which was added either SARS-CoV-2 peptide pools S1 or S2 (2ug/ml, 18-mers with 10 amino acid overlap, Mimotopes) or PMA/Ionomycin as a positive control (2ul/ml, Cell Activation cocktail, Biolegend, 423301); DMSO was used as the negative control at the equivalent concentration to the peptides. Degranulation marker anti-CD107a-BB700 antibody (1:50, clone H4A3, BD Bioscience, 566558) was added into all wells. Cells were incubated at 37^o^C for one hour. Brefeldin A (2ug/ml, GolgiPlug, BD Bioscience, 555029) was added for additional 5 hours into all conditions. Six hours after stimulation cells were prepared for flow cytometry.

***Flow Cytometry of stimulated cells***

Stimulated PBMC were washed with PBS, and cell surface stained with viability dye Zombie Yellow (1:200, Biolegend, 423104) for 30min at room temperature. Cells were washed with PBS/2% FCS, fixed with 4% paraformaldehyde for 20min at RT (ThermoFisher Scientific, 28908), washed and kept at 4^o^C overnight in PBS/2% FCS. Subsequently, cells were washed with PBS, permeabilized with Perm/Wash buffer (BD Biosciences, 554723) according to manufacturer’s instruction, and stained with antibodies (Table 1) for 1 hour on ice in the dark: anti-CD3-BUV395 (1:50, clone UCHT1, BD Biosciences, 563546), anti-CD4-BV785 (1:100, clone SK3, Biolegend, 344642), anti-CD8-BUV496 (1:100, clone RPA-T8, BD Biosciences, 564804), anti-IFNγ-APC (1:25, Miltenyi Biotec, 130-090-762), anti-TNFα-AF700 (1:50, clone MAb11, Biolegend, 502928), anti-IL2-BV421 (1:100, clone 5344.111, BD Biosciences, 562914) and anti-CD154-BV605 (1:50, clone 24-31, Biolegend, 310826). Cells were washed, transferred to flow cytometry 5 ml tubes, and acquired on a Symphony A5 flow cytometer (Beckman Coulter). Data were analysed using FlowJo V10 (BD Biosciences).

**Supplementary table 1.1 List of anti-human flow cytometry antibodies used for T-cell stimulation assay.**

| **Anti-human Antibody (antigen and fluorophore)** | **Clone** | **Dilution** | **Vendor** | **Catalog no.** |
| --- | --- | --- | --- | --- |
| CD107a BB700 | H4A3 | 1:100 | BD Biosciences | 566558 |
| Zombie Yellow Viability Dye |  | 1:200 | Biolegend | 423104 |
| CD3 BUV395 | UCHT1 | 1:50 | BD Biosciences | 563546 |
| CD4 BV785 | SK3 | 1:100 | Biolegend | 344642 |
| CD8 BUV496 | RPA-T8 | 1:100 | BD Biosciences | 564804 |
| IFNγ APC |  | 1:25 | Miltenyi Biotec MACS | 130-090-762 |
| TNFα AF700 | MAb11 | 1:50 | Biolegend | 502928 |
| IL2 BV421 | 5344.111 | 1:100 | BD Biosciences | 562914 |
| CD154 BV605 | 24-31 | 1:50 | Biolegend | 310826 |

Supplementary Data 2

**Supplementary Table 2.1**

| Day post HSCT | Sample type* | PCR assay** | PCR target 1  CT value | PCR target 2  CT value | Composite SARS-CoV-2 RNA result |
| --- | --- | --- | --- | --- | --- |
| +93 – +210 | N&T swabs x 4 | 1 or 2 | Negative | Negative | Not detected |
| +85 – +184 | ET aspirate x 8 | 1 or 2 | Negative | Negative | Not detected |
| +84 | N&T swab | 1 | Negative | Negative | Not detected |
| +78 | N&T swab | 3 | 31.54 | 33.18 | Detected |
| +77 | N&T swab | 2 | 26.20 | Negative | Indeterminate |
| +74 | ET aspirate | 1 | Negative | Negative | Not detected |
| +61 | ET aspirate | 1 | 26.17 | 26.34 | Detected |
| +65 | N&T swab | 3 | 25.46 | 26.22 | Detected |
| +63 | ET aspirate | 2 | 32.09 | Negative | Indeterminate |
| +63 | N&T swab | 1 | 37.93 | Negative | Indeterminate |
| +56 | N&T swab | 2 | 30.74 | 29.72 | Detected |
| +53 | ET aspirate | 3 | 40 | 40 | Not detected |
| +51 | ET aspirate | 4 | Not applicable | | Not detected |
| +49 | ET aspirate | 4 | Not applicable | | Detected |
| +46 | BAL | 1 | 34.99 | 36.95 | Detected |
| +43 | ET aspirate | 2 | 24.83 | 24.64 | Detected |
| +42 | ET aspirate | 1 | 20.50 | 21.00 | Detected |
| +39 | ET aspirate | 1 | 24.63 | 25.25 | Detected |
| +35 | ET aspirate | 1 | 18.61 | 18.16 | Detected |
| +31 | ET aspirate | 1 | 20.41 | 20.53 | Detected |
| +30 | ET aspirate | 1 | 28.34 | 28.34 | Detected |
| +25 | N&T swab | 3 | 18.61 | 19.43 | Detected |
| +21 | N&T swab | 1 | 16.57 | 17.18 | Detected |
| +21 | ET aspirate | 1 | 29.03 | 29.23 | Detected |
| +20 | BAL | 1 | 27.82 | 28.06 | Detected |
| +16 | BAL | 1 | 29.49 | 30.87 | Detected |
| +15 | ET aspirate | 1 | 25.27 | 25.56 | Detected |
| +11 | N&T swab | 3 | 18.25 | 19.12 | Detected |

*N&T: Nose and throat; ET: Endotracheal; BAL: Broncho-Alveolar Lavage

**PCR assay and respective (gene targets): 1. [Altona RealStar® SARS-CoV-2 RT-PCR Kit 1.0 (S & E) – sample extracted on King Fisher automated nucleic acid extraction system and run on ABI 7500 Fast cycler; 2. [Altona RealStar® SARS-CoV-2 RT-PCR Kit 1.0 (S & E) – sample extracted and run on Roche FLOW system; 3. Cobas® SARS-CoV-2 Test (Orf1a/b & E), run on ROCHE 6800 instrument; 4. Biomerieux: BIOFIRE® Respiratory 2.1 plus Panel (S & M) – this assay does not provide CT value output. NOTE: CT values >40 on both PCR gene targets are considered negative. Any discordant gene targets result is reported as indeterminate.
